# Preparation and Mechanical Properties of Low Carbon Cementitious Materials with Superfine Cement Reverse Filling High-Volume Mineral Admixtures

**DOI:** 10.3390/ma16134814

**Published:** 2023-07-04

**Authors:** Shengnan Xu, Zhishan Xu, Yongsheng Ji

**Affiliations:** 1 Jiangsu College of Engineering and Technology, Nantong 226006, China; xushengnan0829@163.com; 2 Jiangsu Key Laboratory Environmental Impact and Structural Safety in Engineering, School of Mechanics & Civil Engineering, China University of Mining and Technology, Xuzhou 221116, China

**Keywords:** mineral admixture, superfine cement, mineral activity, reverse filling, compressive strength

## Abstract

In order to increase the content of mineral admixtures in cement, this study proposes a method for preparing a high-volume mineral admixture cementitious material (HMAC) using superfine cement as a reverse filling material. Firstly, superfine cement is prepared through mechanical grinding. Then, the activity of mineral admixtures (such as slag and fly ash) is enhanced by mechanical grinding, sulfate activation, and alkali activation methods. Meanwhile, the evolution of HMCM from microstructure to macroscopic mechanical behavior is studied by combining a laser particle size analyzer and a scanning electron microscope. Furthermore, the reverse filling mechanism of superfine cement on mineral admixtures under different activation conditions is proposed. Results show that superfine cement can largely improve the utilization rate of cement clinker and the compressive strength of cementitious materials. In the condition that the compressive strength is not lower than that of the control group (without mineral admixture), the content of mineral admixture can be increased to 50%, 70%, and 90% after mechanical grinding, sulfate activation, and alkali activation, respectively. Analysis indicates that the reverse filling effect of superfine cement is the main reason for improving the density of the HMCM.

## 1. Introduction

Industrial carbon emissions are a topic of global concern [1,2,3,4]. Research shows that the cement industry contributes approximately 7% to the total global carbon emissions [5,6,7]. It is evident that this issue has a detrimental effect on the sustainable development of cement production. In recent years, extensive research has been conducted on the utilization of industrial solid waste, such as fly ash and slag, in construction materials [8,9,10,11,12]. This measure offers significant environmental and economic benefits, including the reduction of CO_2_ emissions and the conservation of natural resources like limestone [13,14]. Consequently, the investigation of using industrial solid waste as a substitute or supplement for cement has emerged as a prominent research focus in efforts to mitigate carbon emissions [15,16,17].

Currently, there are two significant approaches to address the issue of carbon emissions in cement production: the utilization of industrial solid waste for the preparation of geopolymers and their use as mineral admixtures [18,19,20]. Among these, the preparation of geopolymers without cement clinker holds immense potential as a low-carbon alternative to cement [21,22,23]. However, the process of preparing geopolymers is complex, and their performance is unstable, necessitating further comprehensive and in-depth research [24,25,26]. The partial replacement of cement with industrial solid waste, such as fly ash and slag, is widely accepted and employed as a low-carbon strategy by cement manufacturers and concrete mixing plants [27,28,29]. Nevertheless, the low reactivity of these mineral admixtures results in their limited usage [30,31,32,33]. Even so, the mineral admixtures commonly used in cement are still high-quality solid waste.

In order to increase the content, the activity of the mineral admixture is usually increased by grinding [34]. However, due to its dense vitreous structure, the mineral admixture primarily serves as a filler, with volcanic ash providing supplementary effects [35,36]. Therefore, the actual utilization rate of industrial solid waste remains quite low. Similarly, the utilization of clinker in ordinary silicate cement is also inefficient. Ordinary silicate cement, with a specific surface area of about 300 m^2^/kg, has an effective utilization rate of less than 50% for one year [36,37,38]. The part of the cement particle that is not fully hydrated acts only as a microaggregate.

Existing studies [39,40] have shown that the utilization of superfine cement can exceed 80%. Shondeep L. Sarkar [41] pointed out two main drawbacks of refined cement particles, namely, late strength regression and faster setting time. However, these adverse effects of refined cement particles can be mitigated by replacing 20% of the cement with ultrafine fly ash. Dale P. Bentz [42] conducted computer simulations to analyze the strength development of different particle size cements, demonstrating that coarse cement lags behind in strength development compared to fine cement. A.K.H. Kwan [43] further showed in their research that incorporating a small amount of ultrafine cement into the voids of ordinary cement can improve the packing density of the cement, thus reducing the water requirement for filling the voids. Mingjuan Zhou [44] utilized a slurry of ultrafine cement to suppress deformation and damage of surrounding rocks in tunnels.

Taken together, it is evident that superfine cement is an efficient cementitious material for enhancing cement performance. However, current research primarily focuses on the application of high dosages of superfine cement, with mineral admixtures only serving as additives to regulate the properties of superfine cement. Therefore, if the roles of cement and mineral admixtures are interchanged, using superfine cement to fill the voids left by mineral admixtures, it is possible to simultaneously enhance the utilization of cement and mineral admixtures by increasing the reactivity of the mineral admixtures. This approach provides a new pathway for the development of low-carbon cementitious materials.

In this paper, superfine cement was produced through the process of ultra-fine grinding. The low-quality industrial solid waste was activated by mechanical grinding, sulfate activation, and alkali activation. By incorporating superfine cement with industrial solid waste, low-carbon cementitious materials were prepared, ensuring that the resulting compressive strength was not lower than that of the control group (without mineral admixture). Additionally, the impact of superfine cement on the microstructure of HMCM was investigated using a combination of X-ray diffraction (XRD) and scanning electron microscopy (SEM). Furthermore, based on the particle size distribution characteristics of superfine cement, a reverse filling mechanism for the mineral admixture in the presence of superfine cement under different activation conditions was proposed.

## 2. Materials and Methods

### 2.1. Materials

(1) Cement

Ordinary silicate cement has a large particle size and contains a small amount of mineral admixture, which does not meet the fineness requirements in this paper. Thus, superfine cement used in this paper is ground from cement clinker mixed with 5% flue gas desulfurization gypsum (all clinkers appearing later are mixed with gypsum). The performance of ultrafine cement is benchmarked using both P.O 42.5 and P.O 52.5 cement for grinding. Their main specifications meet the requirements of Chinese national standard (GB 175-2020) [45]. Their chemical compositions are shown in Table 1, respectively. Standard sand and tap water are used for testing.

(2) Mineral admixture

Fly ash and slag are used as the mineral admixture of superfine cement. The fly ash is Grade III Fly Ash (GB/T 1596-2017) [46]. The water content is 28.7% (dried when used). Fineness (0.045 mm screen residue) is 32%. The main chemical composition is shown in Table 1.

The slag used in the experiment is S95 granulated blast furnace slag (GBFS) powder (GB/T 18046-2017) [47]. The surface area and specific gravity of GBFS are 416 m^2^/kg and 2.70 g/cm^3^, respectively. Its chemical composition is also shown in Table 1.

(3) Activator

Flue gas desulfurization gypsum (FGD gypsum), sodium sulfate, and alkali activated solution are used as activators for mineral admixtures. FGD gypsum (CaSO_4_) is used to adjust the setting time of superfine cement and to improve the activity of mineral admixtures [48,49,50]. It is an industrial waste with the same main chemical composition as natural gypsum dihydrate. The main chemical composition is shown in Table 1.

The sodium sulfate (Na_2_SO_4_) is technical grade with purity greater than 98%. For alkali-activated solutions, sodium hydroxide (NaOH) and sodium silicate (Na_2_SiO_3_) of 9.65% in Na_2_O, 25.22% in SiO_2_, and 65.13% in H_2_O are used. The modulus of liquid sodium silicate is 2.7 and the concentration is 37 °Bé. Dissolve sodium hydroxide in sodium silicate solution and adjust the modulus of sodium silicate solution to 1.2 to obtain alkali-activated solution [51].

### 2.2. Grinding Method of Raw Materials

**Figure 1:**
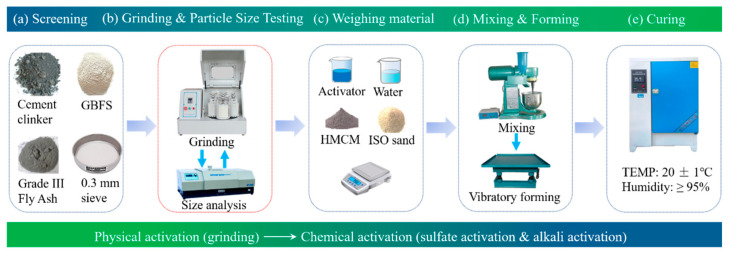
Grinding of raw materials and preparation of specimens.

Superfine cement is prepared by an ultra-fine grinding method in this experiment, with the specific method shown in Figure 1. Firstly, the raw material is screened (0.3 mm mesh) to remove large particles that are difficult to grind. After screening, it is added into the ball mill tank and sealed. Then, the ball mill is started for grinding. At regular intervals, a 5 g sample is taken out and its particle size is measured by a laser particle size analyzer (Winner 3003, Jinan, China). If the particle size meets the requirements of the subsequent test, the grinding is stopped. If not, continue grinding.

The grinding equipment used is a small laboratory vertical planetary ball mill (XQM-4, Hunan, China). The diameters of large, medium, and small balls are 5 mm, 3 mm, and 1 mm, respectively, the number ratio is 4:24:30, the ball material ratio is 1:2, and the ball mill speed is set at 220 r/min.

### 2.3. Preparation of HMCM Mortar Specimens

According to the “Method of testing cement-Determination of strength (ISO Method)” GB/T 17671-1999 [52] to prepare specimens, the specific method is shown in Figure 1. Firstly, mixing water or liquid activator is added to the mixing pot. Then add cementitious materials (superfine cement, mineral admixture) and standard sand in turn and mix well. Finally, it is poured into the mold and formed. The specimen size is 40 mm × 40 mm × 160 mm, and 3 specimens are formed in each pot. Then the molded specimens are placed in a standard curing box at a temperature of 20 ± 1 °C and a humidity of 95%. The specimens are demolded after the age of 1 day, and the compressive strength is measured after the demolded specimens continue to be maintained until the target age.

### 2.4. Research Contents and Methods

#### 2.4.1. Effect of Cement Clinker Fineness on the Compressive Strength of HMCM

(1) Grinding of cement clinker

In this section, separate samples of 5 g each were taken from P.O 42.5, P.O 52.5, and cement clinker. The particle size distribution of these samples was measured using a laser particle size analyzer, serving as a reference for determining the fineness of clinker grinding. Next, three portions of clinker were ground to achieve the fineness grades of P.O 42.5, P.O 52.5, and superfine cement. They were numbered as P.O 42.5′, P.O 52.5′ and SC (superfine cement) in order. The technical index of superfine cement is that the maximum particle size is less than 20 μm and the average particle size is 5 μm (GB/T 35161-2017) [53].

(2) Mix proportion

Ground clinker and fly ash were used as cementitious materials to form mortar specimens. Fly ash replaced 0%, 20%, and 50% of the clinker in equal masses in turn. The liquid–solid ratio was 1/2.5. The cement–sand ratio was 1/3. The specimens were numbered as A-1, A-2, …, A-9 in order. The clinker used in A-1, A-2, and A-3 groups was P.O 42.5′, in A-4, A-5, and A-6 groups, it was P.O 52.5′, and in A-7, A-8, and A-9 groups, it was SC. The compressive strength was measured after standard curing to 3 days/28 days. Meanwhile, the fluidity of mortar was tested. The details of the mortar mix proportions are shown in Table 2.

#### 2.4.2. Effect of Fly Ash Fineness on the Compressive Strength of HMCM

(1) Grinding of fly ash

This section was designed to increase the activity of fly ash by grinding. Specifically, three portions of fly ash were taken, one unground, one ground to the fineness of P.O 52.5 cement, and the last one ground to the fineness close to that of superfine cement, and named unground fly ash (UF), shallowly ground fly ash (SF), and deeply ground fly ash (DF) in turn. The particle size distribution was measured by a laser particle size analyzer after grinding. The particle morphology was observed with an environmental scanning electron microscope (Quanta 250, Hillsboro, OR, USA).

(2) Mix proportion

Superfine cement and fly ash were used as cementitious materials to form mortar specimens. The fly ash replaced 20% and 50% of the superfine cement in equal masses in turn. The liquid–solid ratio was 1/2.5. The cement–sand ratio was 1/3. The specimens were numbered as B-1, B-2, …, B-6 in order. Fly ash used in groups B-1 and B-2 was UF, in groups B-3 and B-4 was SF, and in groups B-5 and B-6 was DF. The compressive strength was measured after standard curing to 3 days/28 days. Meanwhile, the fluidity of mortar was tested. The details of the mortar mix proportions are shown in Table 3.

#### 2.4.3. Effect of Sulfate Activation on the Compressive Strength of HMCM

(1) Sulfate-activated mineral admixtures

This section was designed to improve the activity of mineral admixtures by means of sulfate activation. Since the low calcium content of fly ash was not conducive to the establishment and development of mechanical properties [54], the calcium content of the cementitious material was increased by compounding with slag in this paper. The cementing material was weighed and ground in the ratio of 40:60:6:3 for fly ash: slag: gypsum: sodium sulfate [55,56], and the ground powder was named as H1. The purpose of grinding the sulfate solid powder together with the mineral admixture in this case was to make contact and homogenize it.

(2) Mix proportion

Superfine cement and H1 were used as cementitious materials to form mortar specimens. H1 replaced 0%, 30%, 50%, 70%, and 90% of the superfine cement by equal mass in turn. The liquid–solid ratio was 1/2.5. The cement–sand ratio was 1/3. The specimens were numbered as C-1, C-2, …, C-5 in order. The compressive strength was measured after standard curing to 3 days/28 days. Meanwhile, the fluidity of mortar was tested. The details of the mortar mix proportions are shown in Table 4.

#### 2.4.4. Effect of Alkali-Activated on the Compressive Strength of HMCM

(1) Alkali-activated mineral admixtures

This section was designed to improve the activity of mineral admixtures by means of alkali activation (liquid sodium silicate). The mineral admixture was weighed and ground in the ratio of fly ash: slag as 2:3 [48,50], and the ground powder was named as H2.

(2) Mix proportion

Superfine cement and H2 were used as cementitious materials to form mortar specimens. H2 replaced 90% of the superfine cement with an equal mass. The amount of liquid sodium silicate (measured by solid content) was 0%, 5%, 10%, 15%, and 20% of the cementitious material, respectively. The liquid–solid ratio was 1/2.5 (included water in liquid sodium silicate). The cement–sand ratio was 1/3. The specimens were numbered as D-1, D-2, …, D-5 in order. The compressive strength was measured after standard curing to 3 days/28 days. Meanwhile, the fluidity of mortar was tested. The details of the mortar mix proportions are shown in Table 5.

#### 2.4.5. Effect of Ultrafine Cement on the Microstructure of HMCM

(1) Preparation of paste sample

According to the mix proportions in Section 2.4.3 and Section 2.4.4, the sulphate paste specimens and alkali-activated paste specimens were prepared, respectively. The freshly mixed paste was molded into the test mold 20 mm × 20 mm × 20 mm. After 24 h of standard curing, take off the mold. After demolding, specimens were standard curing to 28 days of age, and then broken into cubic specimens of about 1 cm^3^ volume. Specimens were placed in anhydrous ethanol for 48 h to terminate hydration, and then dried in a constant temperature blast oven at 65 °C for 24 h.

(2) Microstructure test

Small amounts of sulfate and alkali-activated specimens were crushed and ground into powders with particle sizes less than 200 meshes, and their compositions were tested by X-ray diffraction (D8 ADVANCE, Saarbruecken, Saarland, Germany). It was used to study the degree of hydration of mineral admixtures. Sulfate and alkali-activated specimens of relatively regular volume were taken and placed in an ion sputterer for surface gold spraying. Afterwards, the microstructure of the specimens was observed using an ambient scanning electron microscope (Quanta 250, Hillsboro, OR, USA). It was used to study the reverse filling effect of ultrafine cements.

## 3. Results and Discussion

### 3.1. Analysis of the Effect of Cement Clinker Fineness on Compressive Strength

#### 3.1.1. Fineness Analysis

**Figure 2:**
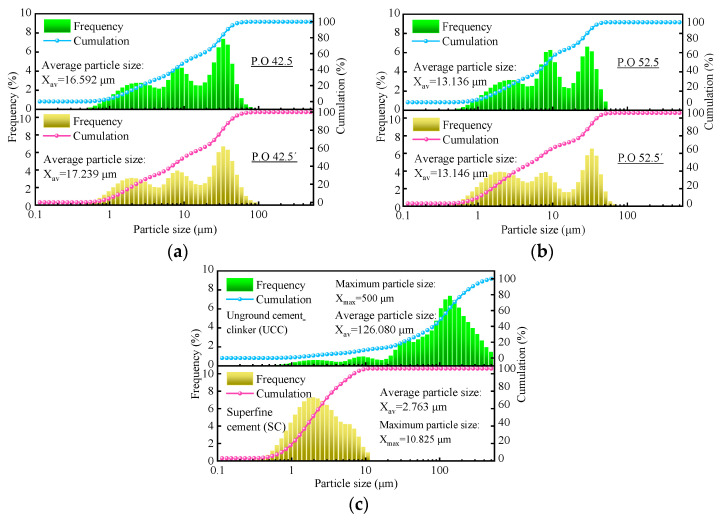
Particle size distribution of cement clinker. (**a**) P.O 42.5′ (Grinding 30 min); (**b**) P.O 52.5′ (Grinding 90 min); (**c**) SC (Grinding 300 min).

The particle size distribution of cement clinker is shown in Figure 2. From the figure, the average particle sizes of the reference samples of P.O 42.5 cement, P.O 52.5 cement, and cement clinker are 16.592 μm (Figure 2a), 13.136 μm (Figure 2b), and 126.080 μm (Figure 2c), respectively. When the grinding time was 30 min, 90 min, and 300 min, the average particle size of cement clinker was 17.239 μm (P.O 42.5′), 13.146 μm (P.O 52.5′), and 2.763 μm (SC), respectively. Particle size distribution of P.O 42.5′ and P.O 52.5′ clinkers are similar to those of P.O 42.5 and P.O 52.5 cement, respectively. The average particle size (X_av_ = 2.763 μm) and the maximum particle size (X_max_ = 10.825 μm) of SC clinker completely meet the technical requirements of superfine cement (X_av_ < 5 μm, X_max_ < 10.825 μm) [39]. Therefore, the materials used in the subsequent tests are P.O 42.5′, P.O 52.5′, and SC, respectively.

#### 3.1.2. Compressive Strength Analysis

(1) 3 days compressive strength

**Figure 3:**
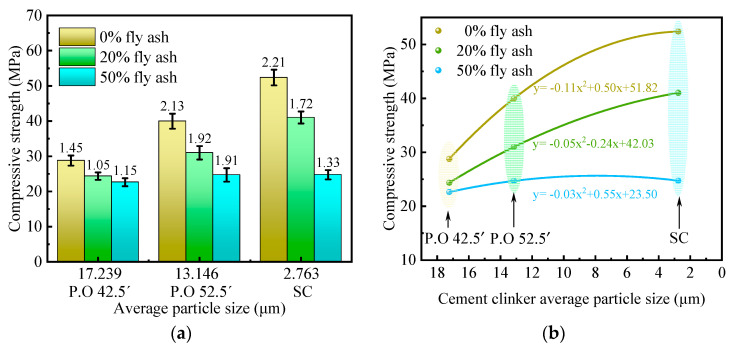
Effect of fineness of cement clinkers on 3-day compressive strength. (**a**) Compressive strength; (**b**) Functional relationship.

Figure 3 shows the effect of cement clinker fineness on 3-day compressive strength. From Figure 3a, for any particle size, the compressive strength of the specimens decreases with the increase of fly ash dosage. It indicates that the activity of fly ash is much lower than that of cement, and it mainly acts as aggregates in the early stages of hydration.

To clearly characterize the relationship between the compressive strength of the specimen and the particle size, the relevant data in Figure 3a are fitted to the image as a function shown in Figure 3b. From the figure, when the content of fly ash is the same, the compressive strength of the specimen increases with the decrease of cement clinker particle size. For example, when the amount of fly ash is 0%, the compressive strength of the SC specimen is 52.38 MPa, which is 1.8 times that of P.O 42.5′ specimen. This indicates that reducing the fineness of cement can greatly improve the early strength. The reason is that the smaller the fineness of the cement, the faster the hydration rate and the more hydration products. This is also on the basis of a small amount of superfine cement mixed with a large number of low-activity mineral admixtures can still have sufficient mechanical strength.

When the dosage of fly ash is 20%, with the decrease of cement clinker particle size, the compressive strength of the specimen gradually increases. Among them, the compressive strength of the SC specimen is 41.01 MPa, which is 1.7 times higher than that of the P.O 42.5′ specimen. It indicates that the superfine cement can effectively improve the dosage of mineral admixture. The reason may be that the superfine cement fully fills the voids between the mineral admixtures. Its hydration product binds the mineral admixture into a whole.

However, when the dosage of fly ash is 50%, the compressive strength of the specimen does not change significantly with the decrease of cement clinker particle size. This indicates that when the mineral admixture is more, the compressive strength cannot be improved by reducing the particle size of cement clinker alone. This may be caused by the fact that the superfine cement is not enough to fill the voids between the mineral admixtures.

(2) 28 days compressive strength

**Figure 4:**
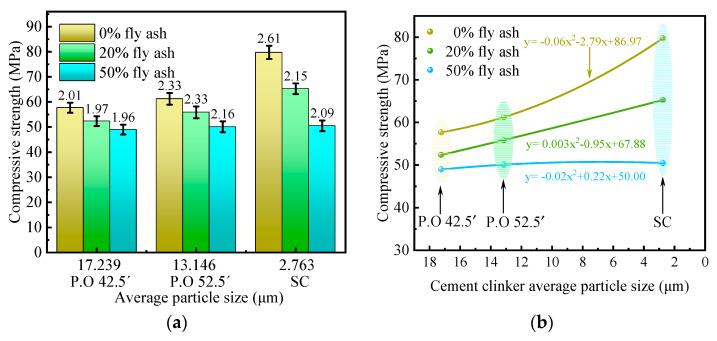
Effect of fineness of cement clinkers on 28-day compressive strength. (**a**) Compressive strength; (**b**) Functional relationship.

As can be seen from Figure 4a,b, the compressive strength of the specimens at 28 days of curing has the same development pattern as the compressive strength at 3 days of curing. When the fly ash dosage is lower than 50%, increasing the fineness of clinker can significantly improve the mechanical strength of the cementitious material. However, when the amount of fly ash reaches 50%, it is not possible to further improve the mechanical strength of the cementitious material by increasing the fineness of clinker only. Therefore, when the amount of mineral admixture is too large, methods are needed to activate the mineral admixture to improve the mechanical strength of the cementitious material.

#### 3.1.3. Mortar Fluidity Analysis

**Figure 5:**
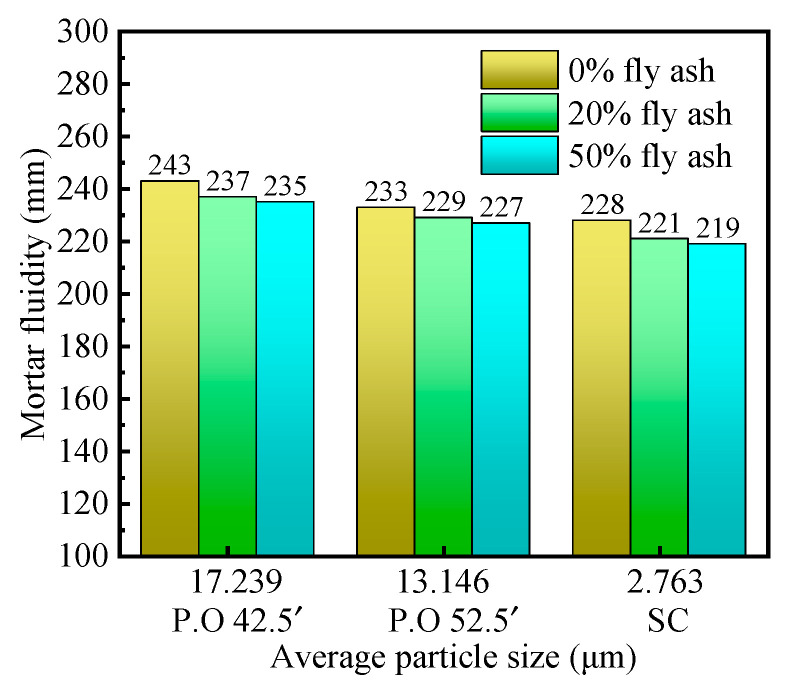
Effect of fineness of cement clinkers on mortar fluidity.

Figure 5 shows the effect of cement fineness on the flowability of mortar. The figure illustrates that when the fly ash content is held constant, the fluidity of the mortar gradually decreases as the fineness of the clinker decreases. This is because finer cement particles have a relatively larger surface area, which enhances their water absorption capacity. As a result, the amount of free water in the mortar decreases, leading to a reduction in flowability. On the other hand, when the fineness of the cement remains constant, the flowability of the mortar also decreases with an increase in fly ash content. This can be attributed to the fact that low-quality fly ash particles have a larger particle size and contain more pores. Therefore, as the fly ash content increases, its water absorption capacity strengthens, causing an increase in mortar viscosity and a decrease in flowability.

### 3.2. Analysis of the Effect of Fineness of Mineral Admixture on Compressive Strength

#### 3.2.1. Fineness and Morphological Analysis

**Figure 6:**
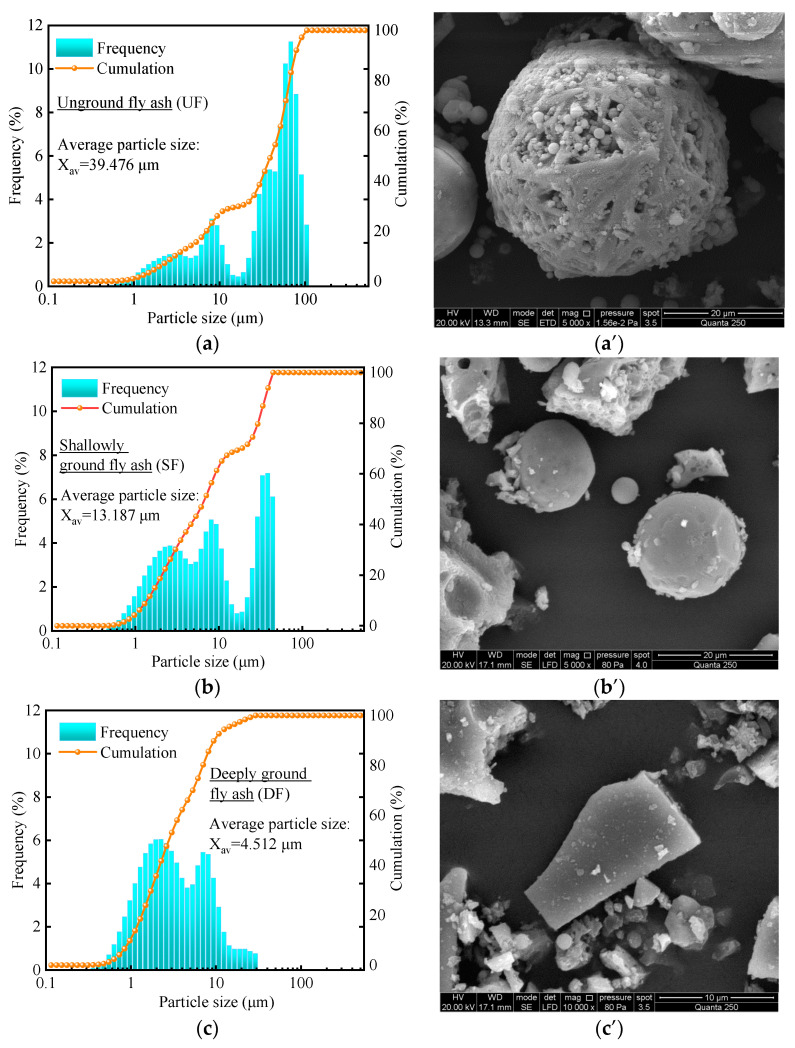
Particle size distribution and morphological changes of fly ash. (**a**) UF; (**a**’) UF—5000×; (**b**) SF; (**b**’) SF—5000×; (**c**) DF; (**c**’) SF—5000×.

Figure 6 shows the particle size distribution and morphological changes of fly ash. Figure 6a shows that the particle size distribution of unground fly ash (UF) is scattered and has more large particles, and the average particle size (X_av_ = 39.476 μm) is much higher than that of P.O 42.5 cement (X_av_ = 16.592 μm), which is commonly used in the cement industry. Figure 6a’ shows that the UF particles are formed by many small particles agglomerated together, and there are a large number of holes and voids inside. Therefore, UF has many defects, and it is difficult to bring out the high mechanical properties.

Figure 6b shows that the large fly ash particles after shallow grinding (30 min) are reduced and the average particle size (X_av_ = 13.187 μm) is close to the level of P.O 52.5 cement (X_av_ = 13.136 μm). This is due to the significant reduction of microscopic defects (Figure 6b’) as the large particles are broken down into numerous dense small particles. It will help the fly ash play a ball effect, which will improve the workability of the cementitious material, as well as reduce the water demand.

Figure 6c shows that the average particle size (X_av_ = 4.512 μm) of fly ash after deep grinding (120 min) is significantly reduced and almost close to the superfine cement grade. This is due to the fact that most of the fly ash particles have been ground and the ball effect of the fly ash particles has been destroyed (Figure 6c’). Additionally, the specific surface area and activity of fly ash particles have been increased.

#### 3.2.2. Compressive Strength Analysis

**Figure 7:**
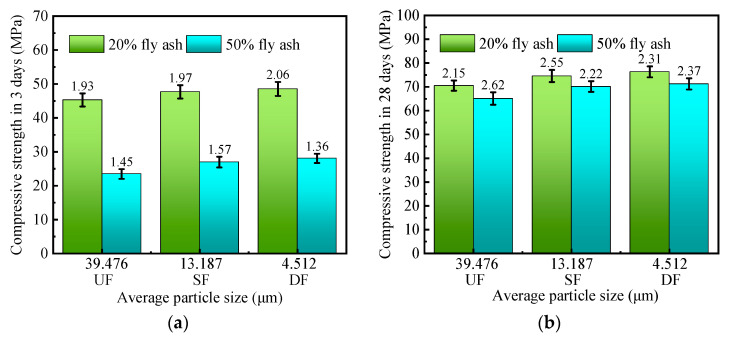
Effect of fineness of fly ash on compressive strength. (**a**) Compressive strength in 3 days; (**b**) Compressive strength in 28 days.

The effect of the fineness of fly ash on the compressive strength is shown in Figure 7. When the specimens are cured for 3 days, with the decrease of fly ash particle size, the increases in compressive strength of the specimens are smaller. Similarly, the compressive strength of the specimens did not increase significantly at 28 days of curing (Figure 7b). This indicates that mechanical grinding does not effectively improve the activity of fly ash. It may be caused by the fact that the main component of fly ash is silicon-aluminum oxide, and the bonding energy of silicon–oxygen bond (Si–O) and aluminum–oxygen bond (Al–O) is high and not easy to break [57,58], resulting in that the fly ash after grinding is still difficult to dissolve. Because the ball effect is beneficial to the workability, the SF is used in the subsequent test.

#### 3.2.3. Mortar Fluidity Analysis

**Figure 8:**
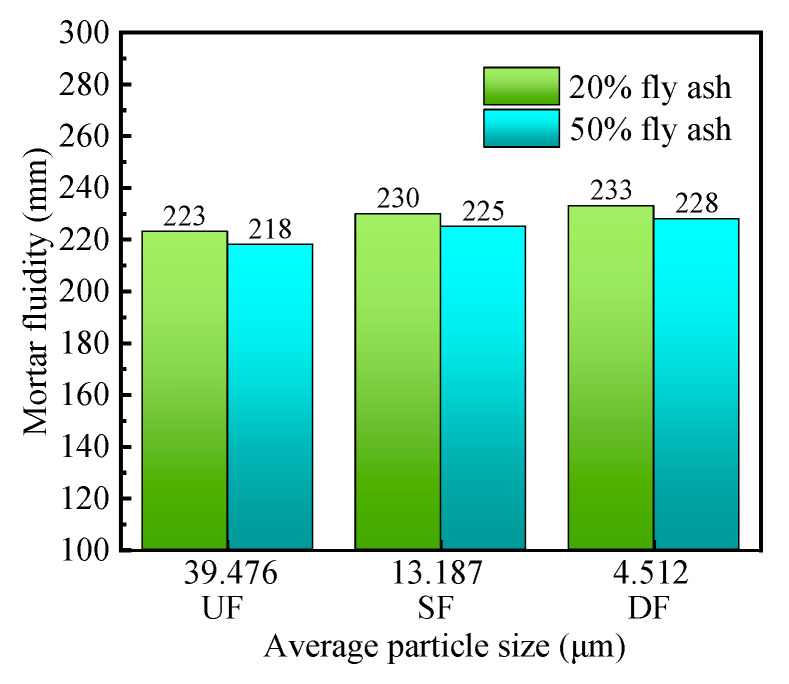
Effect of fineness of fly ash on mortar fluidity.

Figure 8 shows the effect of fly ash fineness on the flowability of mortar. From the graph, it can be observed that when the fly ash content is kept constant, the flowability of the mortar gradually improves as the particle size of the fly ash decreases. This is because unground fly (UF) ash particles have a larger particle size, which hinders the flow of the mortar. However, after grinding, the spherical particles that are aggregated in the larger particles can fully exert their rolling effect. Therefore, as the particle size decreases, the fly ash particles are more easily wetted and flow, leading to a significant improvement in the flowability of the mortar.

### 3.3. Analysis of the Effect of Sulfate on Compressive Strength

#### 3.3.1. Compressive Strength Analysis

**Figure 9:**
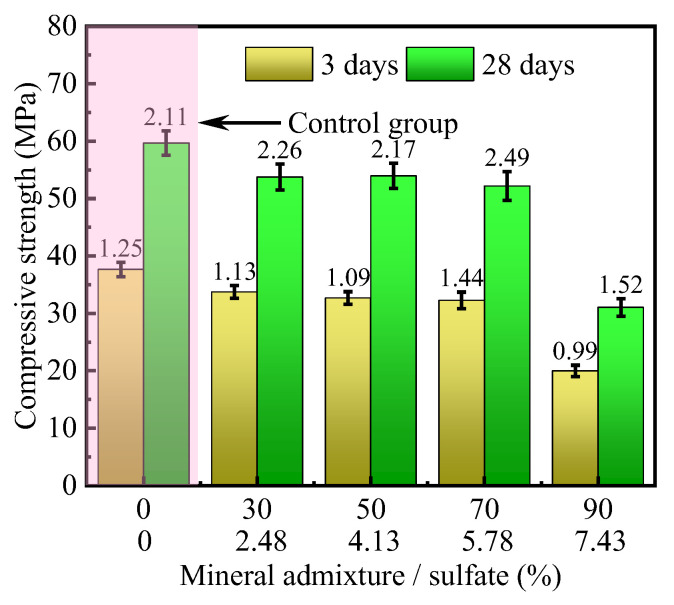
Effect of sulfate on compressive strength.

The effect of sulfate on the compressive strength of HMCM is shown in Figure 9. The amount of sulfate is 8% of the mineral admixture, which is part of the mineral admixture and takes part in the hydration reaction. From the figure, the compressive strength of the specimens at 3 and 28 days of curing shows a trend of slow decrease followed by a sharp decrease with the increase of the mineral admixture (sulfate). When the dosage of the mineral admixture is not more than 70%, the compressive strength of the specimens is always slightly lower than that of the control group with the increase of the dosage.

This indicates that the amorphous glass phase in the mineral admixture (slag, fly ash) is decomposed under the activation of sulfate. Volcanic ash effect is activated and exerts a certain cementing ability. Meanwhile, the superfine cement filled in the gaps of the mineral admixture is fully hydrated. The hydration product further strengthens the bond between the particles of the mixture by powerful cementing ability. Therefore, the compressive strength of the cementitious material did not decrease with the increase of mineral admixture.

When the dosage of mineral admixture is 90%, the compressive strength of the specimens at 3 days/28 days of curing is reduced substantially. This indicates that the dosage of superfine cement is not enough to fill the voids between the mineral admixture particles. The activation of the mineral admixture by sulfate is still not sufficient. Only with a small amount of hydration products of superfine cement is it difficult to cement the mineral admixture dense. Therefore, the compressive strength of the specimens decreased significantly.

#### 3.3.2. Mortar Fluidity Analysis

**Figure 10:**
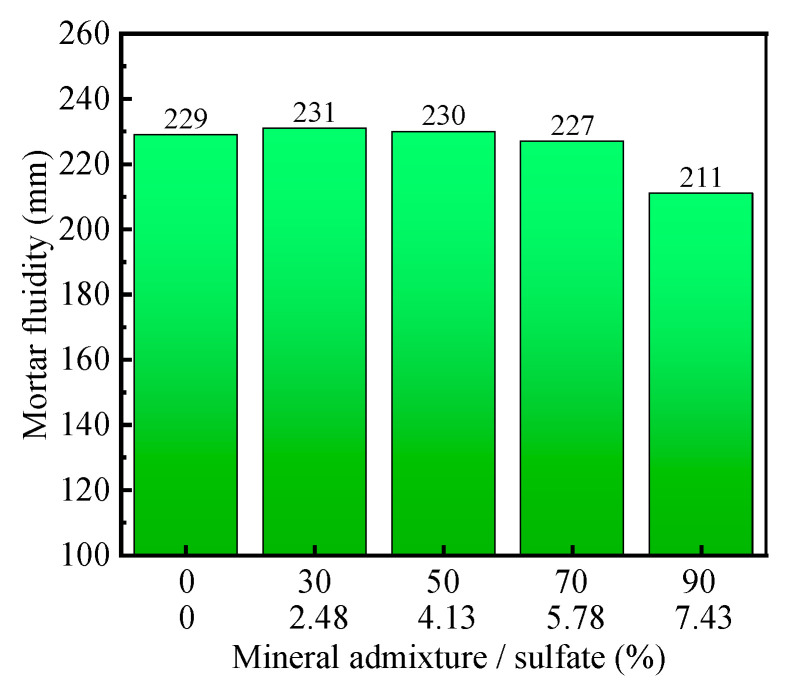
Effect of sulfate on mortar fluidity.

Figure 10 illustrates the effect of sulfate content on the flowability of mortar. From the graph, it can be observed that when the sulfate content is low, there is no significant change in the flowability of the mortar. However, when the sulfate content exceeds 4.13%, there is a noticeable decrease in the flowability of the mortar as the sulfate content increases. This is because the activation effect of sulfates promotes the reaction between mineral admixtures and cement, resulting in an increase in the viscosity of the mortar and a decrease in flowability.

### 3.4. Analysis of the Effect of Liquid Sodium Silicate on Compressive Strength

#### 3.4.1. Compressive Strength Analysis

**Figure 11:**
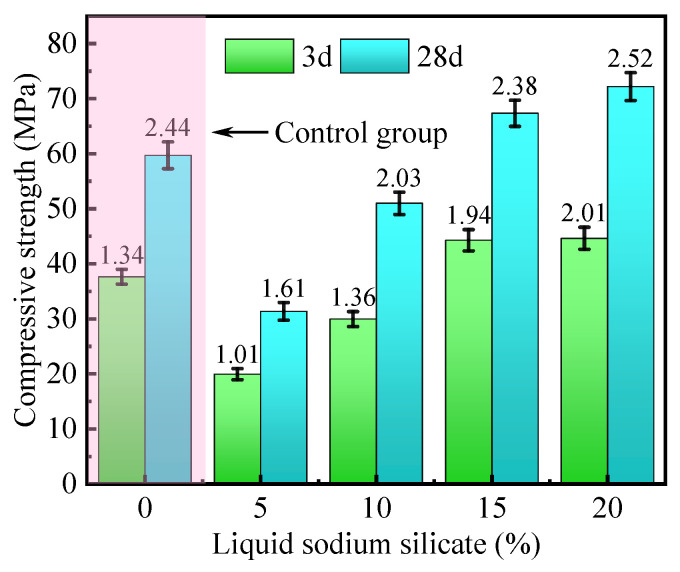
Effect of liquid sodium silicate on compressive strength.

The effect of liquid sodium silicate on the compressive strength of HMCM is shown in Figure 11. It is seen from the figure that the compressive strength of the specimens at 3 and 28 days of curing increased rapidly with the increase in the dosing of liquid sodium silicate from 5% to 20%. When the dosage of liquid sodium silicate is less than 15%, the increase of compressive strength is significant. When the dosage of liquid sodium silicate is more than 15%, the increase of compressive strength is small. When the dosage of sodium silicate is 15%, the compressive strength of the specimens is significantly higher than that of the control group. The target of mechanical strength, after replacing the silicate cement with a large amount of mineral admixture of equal quality that is not lower than that of the control group (without mineral admixture), is achieved.

This indicates that the activity of slag and fly ash gradually increases with the increase of liquid sodium silicate. The reason is that the liquid sodium silicate first transforms to silica gel, releasing hydroxyl (OH^−^). Then OH^−^ breaks the covalent bonds of Si–O–Si, Al–O–Al, and Si–O–Al in fly ash and slag. Broken silica-aluminous material reacts with the calcium component in the liquid phase to form hydrated calcium aluminosilicate and hydrated calcium silicate, which increases the strength of the material. However, when the dosage of liquid sodium silicate is high, the excessive silica content of the paste slows down the dissolution of the mineral admixture, resulting in slower growth of the material strength [57,58]. In summary, the 15% liquid sodium silicate content is most beneficial to the establishment and development of HMCM mechanical strength.

#### 3.4.2. Mortar Fluidity Analysis

**Figure 12:**
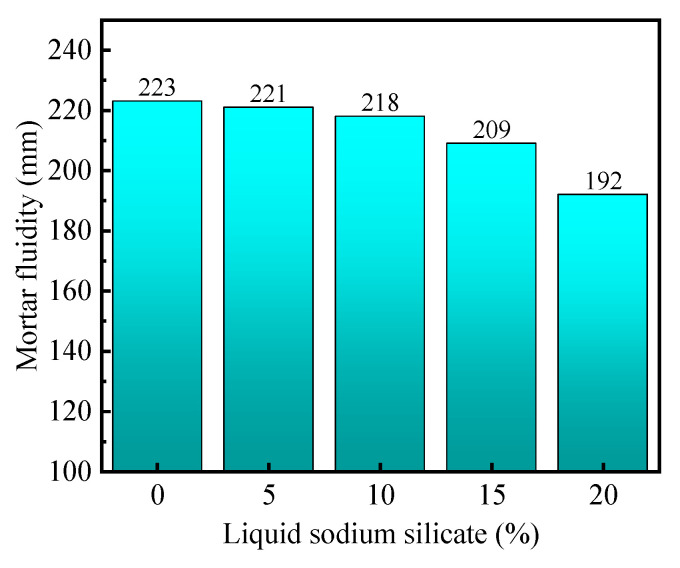
Effect of liquid sodium silicate on mortar fluidity.

Figure 12 illustrates the effect of sodium silicate solution dosage on the flowability of mortar. From the graph, it can be observed that as the dosage of sodium silicate solution increases, the flowability of the mortar gradually decreases, and the decrease in flowability becomes more pronounced with higher dosages. This is because sodium silicate solution is a viscous liquid rich in polymeric sodium silicate, and its viscosity hinders the sliding between mortar particles. Additionally, the high alkalinity of sodium silicate solution promotes the dissolution of mineral admixtures and enhances their reactivity. Therefore, as the dosage of sodium silicate solution increases, the decrease in flowability of the mortar becomes more pronounced.

### 3.5. Analysis of the Effect of Superfine Cement on Microstructure

#### 3.5.1. Analysis of Hydration Products

(1) Sulfate activated specimens

**Figure 13:**
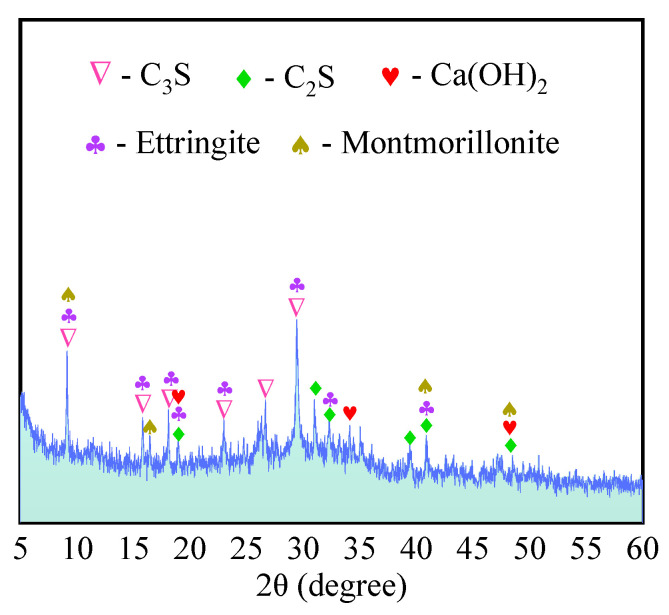
XRD of sulfate-activated specimens (C-4).

The X-ray diffraction pattern of the sulfate activated specimen C-4 (with 70% amount of mineral admixture) at the age of 28 days is shown in Figure 13. As seen in the figure, the diffraction peaks of tricalcium silicate (C_3_S) and dicalcium silicate (C_2_S) have lower peak values. It indicates that specimen C-4 contains cement particles that are not fully hydrated, which may be caused by the presence of cement particles with larger particle sizes in the superfine cement. For example, the superfine cement used in this paper still contains some cement particles with a particle size of about 10 μm. This indicates that even the superfine cement is not completely hydrated in a short time. The diffraction peaks of calcium hydroxide (Ca(OH)_2_) are also low, indicating that the mineral admixture consumed some of the calcium hydroxide by the “volcanic ash effect” [59,60].

However, the diffraction peaks of ettringite and montmorillonite are obviously stronger. It indicates that the activity of the mineral admixture is activated under sulfate activation and higher strength hydration products are generated. It is noteworthy that Figure 9 contains many low diffraction peaks, which indicate that a large amount of amorphous material is generated from the specimen after sulfate activation.

(2) Alkali-activated specimens

**Figure 14:**
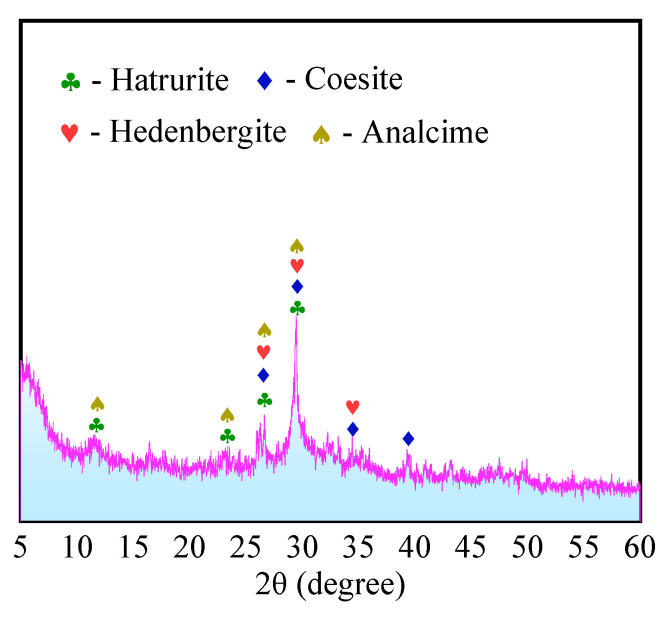
XRD of liquid sodium silicate-activated specimens (D-4).

The X-ray diffraction pattern of alkali-activated specimen D-4 (with 15% amount of liquid sodium silicate) at the age of 28 days is shown in Figure 14. As seen in the figure, there is no longer a diffraction peak of silicate in the specimen. This indicates that the superfine cement is almost completely hydrated. However, the specimen contains a large amount of composite reaction products of aluminosilicate and calcium silicate, such as hatrurite, analcime, coesite, and hedenbergite. These materials are insoluble minerals, which not only have a complete island structure and chain structure similar to organic polymers, but also can form chemical bonds with silica-oxygen tetrahedra and aluminum-oxygen tetrahedra on the surface of mineral particles by dehydroxylation [61]. As a result, the microstructure of HMCM is denser and the mechanical strength is greatly improved.

#### 3.5.2. Micromorphological Analysis

(1) Sulfate activated specimens

**Figure 15:**
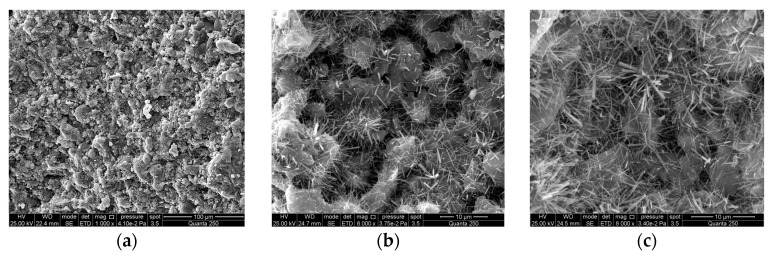
Sulfate-activated specimens (C-4). (**a**) 1000×; (**b**) 6000×; (**c**) 8000×.

The microstructure of the sulfate-activated specimen (C-4) at 28 days of age is shown in Figure 15. Figure 15a shows that the microstructure of the hardened paste is not very dense. However, a large number of gels cement the incompletely hydrated mineral admixture particles into a porous matrix. The particles embedded in the matrix are more uniformly distributed, which makes the hardened paste still have a high mechanical strength. From Figure 15b,c, it can be seen that a large number of fibrous C-S-H gels exist between the particles wrapped and connected by the hydration products. It indicates that the superfine cement acts as a reverse filler and connects the mineral admixture particles using hydration products. However, there are still a large number of unfilled fine pores between the gels. Perhaps these pores can be effectively eliminated only after the mineral admixture is fully hydrated.

(2) Alkali-activated specimens

**Figure 16:**
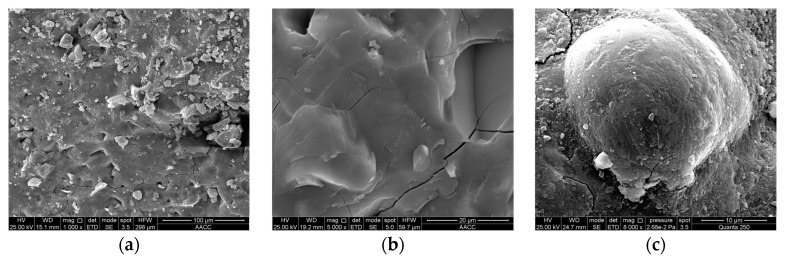
Liquid sodium silicate-activated specimens (D-4). (**a**) 1000×; (**b**) 5000×; (**c**) 8000×.

The microstructure of the alkali-activated specimen (D-4) at the age of 28 days is shown in Figure 16. As can be seen in the figure, the microstructure of the specimens is relatively dense (Figure 16a), but there are a few cracks and a large number of bumps on the surface (Figure 16b). It is due to the complexity of the cementitious material components and the inconsistent hydration rate of different raw materials. In alkaline conditions, the superfine cement distributed in the voids of the mineral admixture particles dissolves rapidly. The generated hydration products bond the mineral admixture particles together and form the primary microstructure.

However, the dissolved silica-alumina material of the mineral admixture and the hydration products of cement continue to generate new hydration products, resulting in the destruction and reconstruction of the primary microstructure and the formation of secondary microstructure. Since the alkali-activated paste is relatively thick, generated hydration products are tightly connected. When mineral admixture particles continue to hydrate after the paste is cured, the microstructure is prone to stress redistribution, resulting in cracks in the dense microstructure [62,63].

Figure 16c shows that there is a large amount of hydration products on the surface of fly ash particles, and they are closely bound to the surrounding materials. This indicates that the “volcanic ash reaction” of fly ash has been achieved, which is also a microscopic factor for the improvement of the strength of HMCM. In summary, liquid sodium silicate can fully activate the industrial waste slag such as slag and fly ash. Therefore, it is practicable to prepare high performance cementitious materials with superfine cement and large amounts of industrial waste slag under alkali-activated conditions.

## 4. Mechanism Analysis

### 4.1. Filling Mechanism of Mineral Admixture

**Figure 17:**
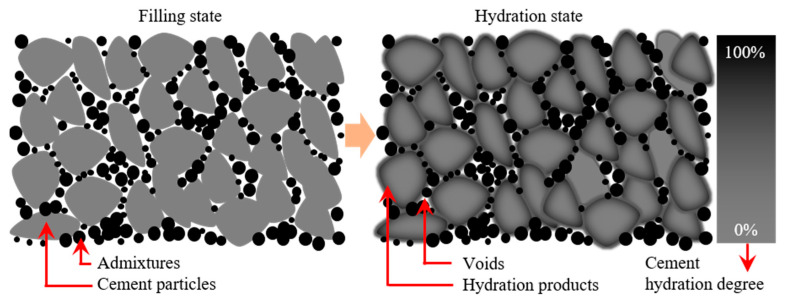
Filling mechanism of mineral admixtures.

Figure 17 shows the filling mechanism of the mineral admixture. As seen in the figure, the particle size of the mineral admixture is much smaller than that of ordinary cement particles, which can fill in the voids between cement particles to increase the density of concrete. However, the voids between cement particles are limited. It is difficult to improve the amount of mineral admixture. It is because the activity of the mineral admixture is low before activation, and to increase the amount of mineral admixture is not good for the establishment and development of the mechanical strength of the cementitious material. Similarly, cement particles with a large particle size are less hydrated and waste a lot of cement resources. Moreover, alkalinity provided by the cement paste is low, which makes it difficult to activate the potential activity of the mineral admixture. Therefore, most of the mineral admixtures only play the role of filler and are not fully utilized.

### 4.2. Reverse Filling Mechanism of Superfine Cement

**Figure 18:**
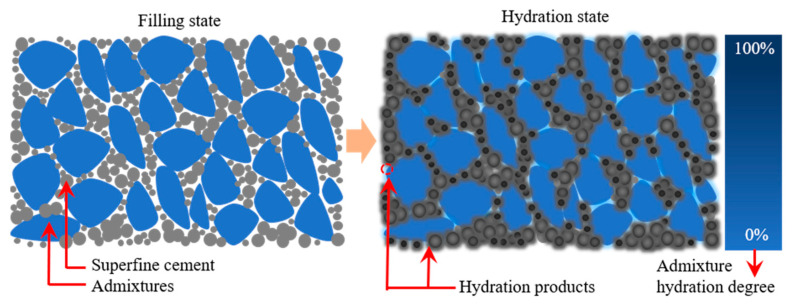
Reverse filling mechanism of superfine cement.

Figure 18 shows the reverse filling mechanism of superfine cement. As seen in the figure, the particle size of superfine cement is much smaller than that of the mineral admixture, which can better fill in the gaps of the mineral admixture and improve its density. Meanwhile, superfine cement has a small particle size and fast hydration rate, which can be fully hydrated in a relatively short time. Therefore, cement clinker is ground to make its particle size smaller than that of the mineral admixture, which has the effect of reverse filling the void of the mineral admixture particles, as well as improve the hardening paste density and the effective utilization of cement clinker.

Superfine cement can be fully hydrated in a short time and generate a large amount of hydration products. Therefore, the content of superfine cement has a great influence on its mechanical properties. When the superfine cement is not enough to fill the voids between the mineral admixtures, its hydration products are also difficult to bind all mineral admixtures tightly. Therefore, the mechanical properties of the hardened paste are low. When the superfine cement is just enough to fill the voids between the mineral admixtures, its hydration products can also bind all the mineral admixtures together. Therefore, the mechanical properties of the hardened paste can be fully improved. When the content of superfine cement exceeds the demand of the voids between the mineral admixtures, the mechanical properties of the hardened paste will be greatly improved and develop towards ultra-high performance.

In summary, the reverse filling effect of superfine cement has the following four main effects: improving the paste density; improving the degree of hydration of cement particles; increasing the utilization rate of cement clinker significantly; and increasing the amount of mineral admixture significantly. Among them, the increase of solid waste mineral admixture not only depends on the reverse filling effect of superfine cement, but also on the activity of the mineral admixture itself. Therefore, improving the activity of the mineral admixture based on the reverse filling effect of superfine cement is another important way to improve the mineral admixture content.

### 4.3. Sulfate Activation Principle Based on Reverse Filling Action

Mineral admixtures usually have a dense glass structure, which makes it difficult to activate their potential activity by grinding. Sulfate can destroy the dense glass structure by reacting with alumina (Al_2_O_3_) and calcium oxide (CaO) in the glass body. Therefore, sulfate has the effect of activating the mineral admixture. However, sulfate is not enough to decompose the glass body sufficiently, and a combination of a certain alkaline environment (pH > 12) is required to promote the dissolution of the other major component of the glass body (SiO_2_) [64,65].

**Figure 19:**
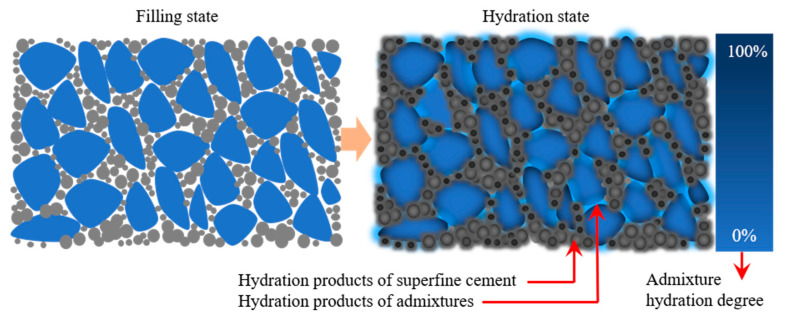
Reverse filling and sulfate activation.

The principle of sulfate activation based on reverse filling action is shown in Figure 19. It can be seen from the figure that the superfine cement improves the density of the matrix. The hydration of the mineral admixture increases with sulfate activation. The reaction process of sulfate activation is shown in Equation (1) [66].

(1) $${\mathrm{Al}}_{2}O_{3}+3\mathrm{Ca}{\left(\mathrm{OH}\right)}_{2}+3\left({\mathrm{CaSO}}_{4}\cdot 2H_{2}O\right)+23H_{2}O\;=3\mathrm{CaO}\cdot {\mathrm{Al}}_{2}O_{3}\cdot 3{\mathrm{CaSO}}_{4}\cdot 32H_{2}O$$

In Equation (1), Al_2_O_3_ is from the mineral admixture and cement, Ca(OH)_2_ is from hydration products of cement, and CaSO_4_·2H_2_O is from desulfurization gypsum. The hydration product is ettringite. From Equation (1), it can be seen that the main reason for sulfate to improve the activity of mineral admixture is to promote the dissolution of Al_2_O_3_. Both ettringite and hydrated calcium silicate have a cementation effect, which promotes the establishment of mechanical properties of HMCM.

### 4.4. Alkali-Activated Principle Based on Reverse Filling Action

The activation effect of sulfate on the mineral admixture depends on a certain alkaline environment. However, the low solubility of calcium hydroxide does not substantially increase the alkalinity of the paste. In contrast, the low modulus liquid sodium silicate not only provides high alkalinity but is also commonly used cementitious material itself.

**Figure 20:**
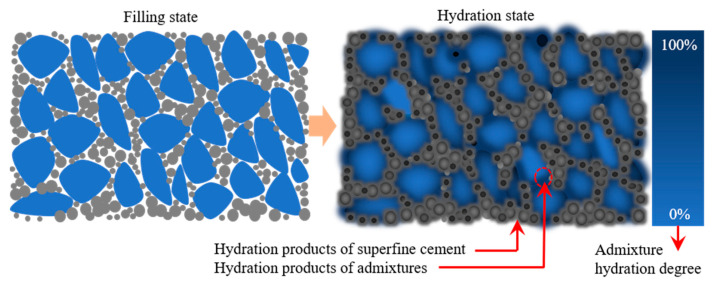
Reverse filling and liquid sodium silicate activation.

The principle of alkali-activated based on reverse filling action is shown in Figure 20. When the liquid sodium silicate makes contact with superfine cement mixture, it first transforms to silica gel and releases a large amount of OH^−^. The OH^−^ penetration into the glass body of the mineral admixture causes covalent bonds such as Si–O–Si, Al–O–Al, and Si–O–Al to break. The breakage of the chemical bond promotes the dissolution of the mineral admixture. The dissolved silica-alumina component reacts with the calcium component in the liquid phase to produce a large amount of hydration products such as hydrated calcium aluminosilicate. It forms a three-dimensional network structure different from the traditional structure of hydrated calcium silicate. The two structures interweave, resulting in a significant increase in the density and mechanical properties of the hardened cement paste.

## 5. Conclusions

1. Increasing the clinker fineness can significantly improve the mechanical strength of HMCM. Superfine cement can reverse fill the voids between the mixture particles and improve the density of the matrix.
2. The low-quality fly ash after grinding does not greatly improve the mechanical properties of the HMCM but facilitates an improvement in the workability of the mortar.
3. The particles with larger size in low-quality fly ash are not dense spheres, but porous aggregates composed of many fine particles.
4. Sulfate has the effect of increasing the activity of mineral admixtures. The amount of mineral admixture in HMCM can be increased to 70% under the condition that the compressive strength is not less than P.O 42.5 cement.
5. Liquid sodium silicate can significantly improve the activity of mineral admixtures. The amount of mineral admixture in HMCM can be increased to 90% under the condition that the compressive strength is not lower than that of P.O 42.5 cement.
6. The reverse filling effect of superfine cement has four functions. First, improve the density of the paste. Second, improve the hydration degree of cement particles. Third, significantly improve the utilization rate of cement clinker. Fourth, significantly improve the amount of solid waste admixture.
7. The reverse filling of the mineral admixture with superfine cement has the potential to enhance the utilization of both cement and mineral admixture, thereby facilitating the preparation and development of low-carbon cementitious materials. However, further in-depth and comprehensive research is still required to explore the preparation of superfine cement and enhance the activity of mineral admixture.

## Figures and Tables

**Table 1:** Chemical compositions of raw materials (wt. %).

Category	Cement Clinker	P.O 42.5	P.O 52.5	FGD Gypsum	Fly Ash	GBFS
SiO_2_	22.22	23.78	22.41	2.76	50.43	31.35
A1_2_O_3_	4.36	4.75	4.38	0.75	32.53	18.65
Fe_2_O_3_	3.13	3.38	3.22	0.65	3.36	0.57
CaO	65.10	56.75	58.5	32.6	5.36	34.65
MgO	1.27	2.34	2.11	1.21	1.09	9.31
Na_2_O	0.66	0.21	0.44	-	-	0.36
K_2_O	0.68	0.78	1.02	-	-	0.45
SO_3_	0.69	2.31	2.48	42.9	-	-
LOI	0.52	2.12	2.03	19.23	5.65	0.71

**Table 2:** Effect of cement clinker fineness on the compressive strength (g).

Category	A-1	A-2	A-3	A-4	A-5	A-6	A-7	A-8	A-9
Fly ash	-	90	225	-	90	225	-	90	225
P.O 42.5′	450	360	225	-	-	-	-	-	-
P.O 52.5′	-	-	-	450	360	225	-	-	-
SC	-	-	-	-	-	-	450	360	225
Water	180	180	180	180	180	180	180	180	180
Sand	1350	1350	1350	1350	1350	1350	1350	1350	1350

P.O 42.5′: Clinker with similar particle size to P.O 42.5; P.O 52.5′: Clinker with similar particle size to P.O 52.5; SC: Superfine cement.

**Table 3:** Effect of fly ash fineness on the compressive strength (g).

Category	B-1	B-2	B-3	B-4	B-5	B-6
SC	360	225	360	225	360	225
UF	90	225	-	-	-	-
SF	-	-	90	225	-	-
DF	-	-	-	-	90	225
Water	180	180	180	180	180	180
Sand	1350	1350	1350	1350	1350	1350

UF: Unground fly ash; SF: Shallowly ground fly ash; DF: Deeply ground fly ash.

**Table 4:** Effect of sulfate activation on the compressive strength (g).

Category	C-1	C-2	C-3	C-4	C-5
SC	450	315	225	135	45
H1	-	135	225	315	405
Water	180	180	180	180	180
Sand	1350	1350	1350	1350	1350

H1: The cementing material was weighed and ground in the ratio of 40:60:6:3 for fly ash: slag: gypsum: sodium sulfate.

**Table 5:** Effect of alkali-activated mineral admixtures on the compressive strength (g).

Category	D-1	D-2	D-3	D-4	D-5
10% SC + 90% H2	450	450	450	450	450
Sodium silicate solution	-	52.3	104.7	157.0	209.3
Water	180	150.2	120.3	90.5	60.7
Sand	1350	1350	1350	1350	1350

H2: The cementing material was weighed and ground in the ratio of 2:3 for fly ash: slag. Sodium silicate solution: Modulus 1.2, with 43% solids and 57% moisture.

## Data Availability

Not applicable.

## Abbreviations

The definition of terms.

HMCM	High-volume mineral admixture cementitious material
FGD gypsum	Flue gas desulfurization gypsum
SC	Superfine cement
UF	Unground fly ash
SF	Shallowly ground fly ash
DF	Deeply ground fly ash
